# Considering Family Dog Attachment Bonds: Do Dog-Parent Attachments Predict Dog-Child Attachment Outcomes in Animal-Assisted Interventions?

**DOI:** 10.3389/fpsyg.2020.566910

**Published:** 2020-08-31

**Authors:** Shelby H. Wanser, Amelia Chloe Simpson, Megan MacDonald, Monique A. R. Udell

**Affiliations:** ^1^Department of Animal and Rangeland Sciences, Oregon State University, Corvallis, OR, United States; ^2^College of Physical Activity and Sport Sciences, West Virginia University, Morgantown, WV, United States; ^3^College of Public Health and Human Sciences, Oregon State University, Corvallis, OR, United States

**Keywords:** human-animal interaction, animal-assisted intervention, Do As I Do, attachment, Secure Base Test, dog, family, children

## Abstract

Animal-Assisted Interventions (AAI) have become more prevalent in recent years, with dog-assisted interventions among the most popular. The literature suggests that a variety of dog-human interventions have the potential for beneficial outcomes for human participants and owners, however, critical gaps in knowledge still exist. Research addressing intervention outcomes for dogs, and the impact of AAI on the dog-human bond, has lagged behind. Even less is known about how dogs perceive child partners in AAI settings. The current study, which involved AAI for youth with developmental disabilities and their family dog, aimed to determine if the dog’s style of attachment to a primary adult caretaker in the home was predictive of dog-child attachment style pre-and post-intervention. Using a Secure Base Test (SBT), the attachment style of the family dog toward an adult owner/parent was evaluated, and the attachment style of the dog toward the participating child was assessed before and after the dog-assisted interventions. The dog’s attachment style to the child was then compared to the dog-parent attachment style. The findings show that all dogs with a secure attachment to the child at the initial assessment also had a secure attachment to the parent. It was also demonstrated that AAI has the potential to change the attachment style between a family dog and child to a more secure attachment, and that the dog-parent attachment style is a significant predictor of which dogs were able to develop a secure attachment to the child over the course of the AAI.

## Introduction

Animal-Assisted Interventions (AAI) have increased in prevalence in recent years (Julius et al., 2013; O’Haire, 2017). Consequently, there has been increased research on the effectiveness and efficacy of different AAI approaches and predictors of outcomes across different populations (O’Haire, 2017; Hu et al., 2018; Jones et al., 2019; Chitic et al., 2020). Dog-assisted interventions are among the most common AAI, likely due to a number of factors including, but not limited to, a dog’s accessibility, trainability, cost of care, and size (Linder et al., 2018). Critical gaps in knowledge about factors that may lead to successful AAIs still exist (O’Haire, 2017; Wanser and Udell, 2019; Wanser et al., 2019; Chitic et al., 2020). For example, little research has focused on the dog’s perception of, or response to, the human participant or the intervention experience. This factor could play an important role in the efficacy of the dog’s performance in the intervention and have implications for the wellbeing of both the dog and the human. How the dog perceives and responds to the human participant may be especially important for AAIs designed for children, including children with disabilities (Wanser et al., 2019). Animals, including dogs, have been known to respond in less predictable ways in the presence of these populations which can in some cases lead to increased risk (Overall and Love, 2001; Yin, 2011). Nevertheless, dogs are commonly used in therapeutic settings and interventions with both children and individuals with disabilities (Leonardi et al., 2017; Jones et al., 2019). Less is known about why some dog-child pairs clash (e.g., specific stimuli, strained interaction history, etc.) and others form successful relationships in home and/or intervention settings.

One factor that may be particularly relevant is attachment. Attachment can be defined as a bond that forms between two individuals, often a dependent individual (child or animal) and their caregiver, that promotes contact- and proximity-seeking, as well as stress reduction and facilitation of independent behavior in the case of secure attachments (Bowlby, 1958; Harlow, 1958). Research has demonstrated that dogs can form attachment bonds to their human caregivers (Topál et al., 1998; Palmer and Custance, 2008; Mariti et al., 2013) and humans can form attachment bonds to their dogs (Barker and Barker, 1988; Cohen, 2002; Kurdek, 2009). Once established, these bonds have the potential to benefit both the animal (Serpell and Barrett, 1995) and human with the strength and quality of attachment (e.g., attachment style) serving as predictive variables for health and welfare outcomes (Garrity et al., 1989; Rooney and Bradshaw, 2002; Bennett and Rohlf, 2007; Meyer and Forkman, 2014; Wanser et al., 2019). Furthermore, it is possible that the influence of AAI’s conducted with a participant’s own pet could be impacted by the nature and strength of the pre-established bond between the participant and animal (Wanser et al., 2019), or that participation in an AAI could alter the quality of the dyad’s attachment bond, potentially in both the AAI and home settings.

When considering attachment quality, a range of different styles of attachment have been identified, which can broadly be divided into secure and insecure attachment styles. Individuals with secure attachment bonds can more effectively use their caretaker to reduce stress and display contact-exploration balance (Secure Base Effect) that allows them to explore and engage effectively in novel contexts and environments (Bowlby, 1958; Julius et al., 2013). Individuals with insecure attachments are still bonded to their caretaker, but this bond does not as readily facilitate stress reduction or a return to normal behavior in novel contexts (Bowlby, 1982; Ainsworth, 1989; Julius et al., 2013). There is currently some evidence that a dog’s attachment style toward their caretaker may influence their performance in AAI contexts (Wanser and Udell, 2019; Wanser et al., 2019). For example, in one Animal-Assisted Activity (AAA) study, the attachment style between a trained therapy dog and their handler/caretaker was evaluated prior to a mock therapy session. Dogs behaved similarly toward their handlers and toward the mock therapy participants independent of attachment style with one exception: Dogs with an insecure attachment to their handler spent more time gazing back at that handler (and consequently less time gazing at the therapy participant) during the session compared to securely attached dogs. Such factors could influence therapeutic outcomes, and could also indicate that securely attached dogs may have lower stress levels during at least some forms of AAA sessions compared to those with insecure attachments (Wanser and Udell, 2019).

Oftentimes dogs who participate in AAI are handled by their owner and engage with unfamiliar AAI participants (as in the abovementioned study), but other times AAI can involve a human participant engaging with a familiar dog, especially in AAI targeted for children and their family pet dog (Tepfer et al., 2017). In such cases, understanding the possible connection between attachment quality and AAI participation could help predict the likelihood of achieving social support and other beneficial outcomes across settings. For example, it has been shown that a human participant’s feelings of attachment toward a dog during AAI promotes participation in the intervention, including greater motivation to attend and greater pro-social engagement (Jones et al., 2019). Thus, one goal of dog-assisted interventions with children might be to establish or promote secure attachments between the dog and child engaged in AAI given the associated benefits reported in cases where stronger attachment relationships are perceived or exist. However, currently, little research exists on dog attachment bonds to children (Wanser et al., 2019). While it has been established that dogs can form attachment to one or more human caregivers (Topál et al., 1998; Gácsi et al., 2001; Parthasarathy and Crowell-Davis, 2006; Mariti et al., 2011, 2013), dogs do not form an attachment, much less a secure attachment, to every human they interact with (Thielke and Udell, 2020). While it is possible that a child in the same household may serve as an attachment figure for a family dog (Wanser et al., 2019), other (non-caregiver attachment) bond types may also be possible. For example, in humans, siblings that engage in caretaking behavior sometimes serve as attachment figures for younger children. However, siblings that do not engage in protective or caregiving behavior typically do not serve as primary attachment figures (Stewart, 1983). Nonetheless, siblings may share other forms of bond (Stewart, 1983). Therefore, it is possible that not all children will serve as a primary attachment figure for a dog in their household, even if they have developed some form of bond. In cases where a caregiver type bond is established between dog and child, the quality of attachment will likely vary between dyads (Wanser et al., 2019). Therefore, it is important to consider the style, or quality, of attachment relationship between dog and child, not just the presence or absence of a bond.

It is common for adults to be the primary caretakers of family dogs (Hall et al., 2016), which may be one reason the majority of work on dog-human attachment has focused on the relationship between dogs and their primary adult owner. However, evidence from the human literature suggests that the establishment of a secure attachment style to a primary caretaker can influence the strength and security of attachments formed with other individuals (Simpson, 1990; Maccoby, 1992; Smyke et al., 2010). Therefore, the attachment style of dog-adult owner pairs may also be a relevant consideration in AAI applications with child participants, as the quality of this primary attachment relationship could potentially predict (1) therapeutic or intervention performance directly (Wanser and Udell, 2019) and/or (2) the likelihood of secure attachment development between dog and child (Simpson, 1990; Maccoby, 1992; Smyke et al., 2010).

To our knowledge, no research to date has compared a dog’s attachment bonds with both adult and child family members within a household. The influences of AAI participation on the dog’s attachment behavior toward a child participant have also not been evaluated. Given that human-dog attachment has been shown to influence both human therapeutic outcomes and dog behavior in AAI settings, when considering AAI with children and family pet dogs, it may also be important to ask how pre-existing relationships between the dog and adult caretaker in the home could influence the dog-child bond and AAI motivation and performance, as well as how AAI influences the dog-human bond.

## Pilot Study

The purpose of this initial study was to (1) evaluate attachment styles between dog-child dyads within an AAI setting across assessment time points to see to what extent secure attachments exist in this setting and (2) determine if a relationship existed between a dog’s attachment style to an adult owner/parent in the household and to the child participant. We predicted that at least some dog-child dyads would display a secure attachment style. However, based on human attachment style research (Smyke et al., 2010), we predicted that dogs showing a secure attachment to an adult owner/parent would be more likely to show or develop a secure attachment to the child participant. As with other pro-social outcomes in prior AAA research (Tepfer et al., 2017), we predicted that the attachment style of dogs would either remain constant or become more secure over the course of AAI participation.

### Methods

#### Participants

Seven youth with developmental disabilities and their parent were recruited to participate in this study with their family dog (see Table 1). Parents completed a demographic questionnaire to indicate their child’s specific disability (no clinical assessments were conducted during the course of the AAI itself).

**TABLE 1:** Participant demographic information for Pilot Study.

**Child participants (*n* = 7)**	
Age (years)	Range = 9–16; Mean = 12.7; SD = 2.9
Sex	Female = 5; Male = 2
Race	White = 6; Asian/Pacific Islander = 1
Primary Disability	Cerebral Palsy = 5; ADHD = 2
**Parent participants (*n* = 7)**	
Sex	Female = 7; Male = 0
**Dog participants (*n* = 7)**	
Age (years)	Range = 1–7; Mean = 3.4; SD = 2.5
Sex	Female = 5; Male = 2
Breed	Labrador Retriever = 2; Labrador Retriever mix = 1; Goldendoodle = 1; Golden Retriever = 1; Chihuahua = 1; Pomeranian = 1

#### Ethical Note

All children, parents, and dogs participated on a voluntary basis. Written informed consent was obtained from the parents/guardians of all participants, and assent was obtained from all of the children explicitly indicating their understanding and desire to participate in the research. The Institutional Review Board (IRB) and Institutional Animal Care and Use Committee (IACUC) of Oregon State University approved all methods and procedures for this study.

#### Intervention

Youth participants, identified as having a developmental disability, and their family dog were recruited from the northwestern region of the United States to participate in an animal-assisted intervention focused on joint physical activity. The AAI consisted of one session per week for 8 weeks, at a veterinary teaching hospital. Weekly AAI activities, led by a trained research assistant, consisted of joint physical activities for the child-dog dyad that were developmentally appropriate and tailored to the child’s skills. For example, children might work on sit-to-stand skills and these were jointly completed by the child and their family dog for 10–15 repetitions. Other activities included jointly balancing on a wobble board, walking, playing catch together, and participating in cavalettis (i.e., small jumps). For homework the child participants were instructed to walk their dog for 30 min and practice the intervention exercises with the dog around the same time every day. This was intended to help establish a bond, create a habitual routine, and foster prompting behavior in the dog (e.g., attention-getting/walk-oriented behavior around the established time). A more detailed description of the intervention methodology, including intervention exercise descriptions has previously been published (please see Tepfer et al., 2017 for a review). Assessments were conducted with the participant-dog dyads before and after the 8-week intervention, as well as 6-months later.

#### Secure Base Test

**FIGURE 1:**
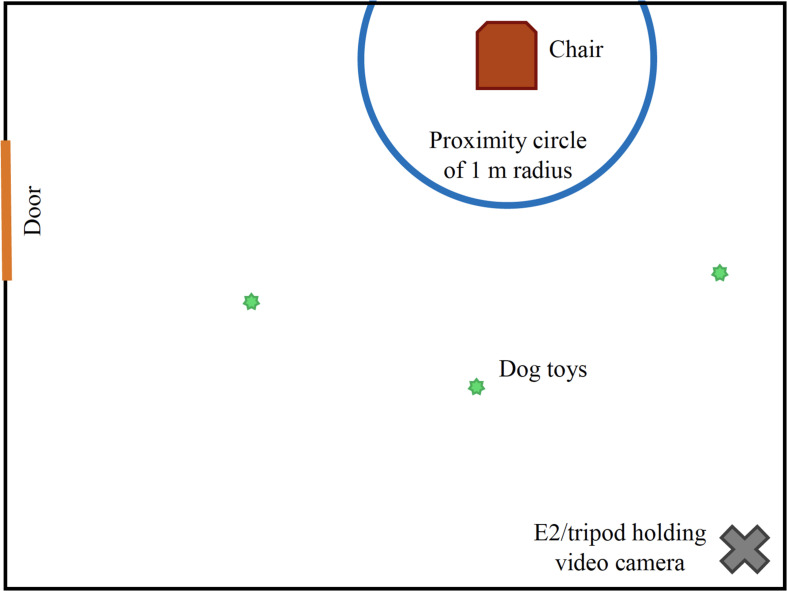
Diagram of room set-up for Secure Base Test.

The Secure Base Test (SBT) was used to evaluate the attachment behavior of the family dog toward both the child participant and adult owner/parent at the initial and follow-up assessments. This test was originally developed to assess the quality of attachment of non-humans to attachment figures (Harlow, 1958) and has been used to evaluate dog-human attachment style and security across multiple settings (Thielke et al., 2017; Thielke and Udell, 2019, 2020), including Animal Assisted Activities (Wanser and Udell, 2019) and was therefore especially well-suited to evaluate attachment style in the current study. Assessments were conducted in a room that was novel to the dog and human participants prior to testing. One chair was located inside a marked circle of 1-m radius on the floor, along a wall adjacent to the door (see Figure 1). Three toys – tennis ball, rope toy, and plush-squeak toy – were on the floor outside the circle. Two experimenters (E1 and E2) conducted the test. E1 provided instructions at the start of each phase to ensure consistent participant behavior (E1 remained outside of the room during all phases). E2 stood neutrally/inattentively in a corner of the room controlling the video camera (except during the alone phase when the camera was left on a tripod). The SBT was divided into three two-minute phases:

##### Baseline/habituation phase

The experimenter led the dog and the human participant (i.e., child or parent) into the room and indicated for them to remove the dog’s leash and sit in the chair. The human participant was instructed that when the dog entered the circle surrounding their chair, they could interact with the dog (i.e., talking/petting/playing), but when the dog was outside the circle, they must remain silent, passive, and non-moving.

##### Alone phase

E1 opened the door to indicate to the human participant to exit the room. E2 left the camera on the tripod filming toward the door and also exited, leaving the dog alone. The alone phase serves as a mild stressor, allowing for assessment of the Secure Base Effect during the return phase.

##### Return/experimental phase

E1 directed the human participant to enter the room and follow the same instructions as the baseline phase. E2 followed closely behind the participant in entering the room and returned to the corner to control the camera, without any interaction with the dog.

#### Behavior Coding

All assessments were video recorded. The return phase was viewed by two coders, with prior training in evaluating canine attachment styles. These two coders independently categorized the dogs’ behavior using an ethogram for canine attachment style categories and definitions previously established in the literature (Schöberl et al., 2016; Thielke et al., 2017; Thielke and Udell, 2019, 2020; Vitale et al., 2019; Wanser and Udell, 2019): secure, insecure ambivalent, insecure avoidant, and insecure disorganized (see Table 2). Inter-rater reliability was then assessed for the full data set (75.7% IRR for attachment style categorization, binomial probability test, *p* < 0.001). After independent IRR analysis, categorization disagreements were then jointly reviewed to come to consensus for the final attachment style designation using the standard procedure for holistic canine attachment style categorization (Thielke et al., 2017; Thielke and Udell, 2019, 2020; Wanser and Udell, 2019). The broader categorization of secure or insecure attachment, indicating the presence or absence of the Secure Base Effect, was the primary focus in this study.

**TABLE 2:** Canine attachment style definitions (adapted from Schöberl et al., 2016 and Thielke et al., 2017).

Attachment Style	Definition
Secure	Dog’s greeting behavior is active, open, and positive. Little or no resistance to contact or interaction with the human participant. Seeks proximity and is comforted upon reunion, returning to exploration or play.
Insecure Ambivalent	Dog shows exaggerated proximity-seeking and clinging behavior (but may struggle if held by human participant). Exhibits a mix of persistent distress with efforts to maintain physical contact with the human participant and/or physically intrusive behavior toward the human participant (Dogs who the judges agreed seemed essentially secure but with ambivalent tendencies were categorized as secure).
Insecure Avoidant	Dog shows little or no visible response to the human participant’s return. Ignores or turns away from human participant but may not resist interaction altogether (e.g., laying, sitting, or standing without physical contact with, out of reach of, or at a distance from human participant).
Insecure Disorganized	Dog exhibits evidence of a strong approach-avoidance conflict or fear upon reunion (e.g., circling human participant, hiding from sight, rapidly dashing away upon reunion, or “aimless” wandering around the room). A lack of coherent strategy is shown by contradictory behavior. Dog may show stereotypies upon reunion (e.g., freezing or compulsive grooming). “Dissociation” may be observed, that is, still or frozen posture, staring into space without apparent cause, for at least 20 s (in a non-resting, non-sleeping dog).
Unclassifiable*	Judges were unable to reach consensus on the attachment style categorization of the dog. Unclassifiable dogs were excluded from further analysis on dog attachment.

*No dogs in the current study were unclassifiable.

### Results and Discussion

The dog-child attachment style changed from an insecure style pre-intervention to a secure style post-intervention in two instances. Two dogs displayed a secure attachment to their child pre- and post-intervention, and the remaining three dogs displayed insecure attachments to the child both pre- and post-intervention. The dog-parent attachment style was a strong predictor of whether a secure attachment style was present or would develop between the dog and child during the intervention (Fisher’s Exact Test, *p* = 0.03). Four dogs were categorized as having a secure attachment to the adult owner/parent, and also had or developed a secure attachment toward the child by the last assessment. The remaining three dogs displayed an insecure attachment to the adult owner/parent and remained insecurely attached to the child throughout the study.

While the population under test in this initial pilot study was small, it still identified statistically significant differences that would suggests that AAI participation has the potential to improve human-animal interactions and, critically, the dog-parent attachment style was a significant predictor of which dogs were able to develop a secure attachment to their child partner during AAI. This is consistent with attachment research in human infants, where individuals with secure attachments to primary caregivers or foster parents facilitated secure attachment development to future attachment figures or adoptive parents (Smyke et al., 2010).

## AAI Experiment

In this AAI Experiment, we sought to explore whether the findings of our pilot study would be generalized in the context of a novel AAI setting with a larger sample size.

### Methods

#### Participants

Twenty-four youth with developmental disabilities and their parent were recruited to participate in this study with their family dog (see Table 3). Parents completed a demographic questionnaire to indicate their child’s specific disability (no clinical assessments were conducted during the course of the AAI itself). Two pairs of siblings participated and shared the same dog and parent between them. Thus, twenty-two pet dogs and twenty-two parents participated in this study.

**TABLE 3:** Participant demographic information for AAI Experiment.

**Child participants (*n* = 24)**	
Age (years)	Range = 8–17; Mean = 11.3; SD = 2.5
Sex	Female = 10; Male = 14
Race	White = 19; Latino/Hispanic = 2; Alaskan Native = 2; unknown = 1
Primary Disability	Autism Spectrum Disorder = 7; ADHD = 5; Intellectual Disability = 4; Down Syndrome = 2; Fetal Alcohol Spectrum Disorders = 2; Anxiety Disorder = 2; Specific Learning Disability = 1; Physical Disability = 1
**Parent participants (*n* = 22)**	
Sex	Female = 17; Male = 5
**Dog participants (*n* = 22)**	
Age (years)	Range = 0.4–10; Mean = 3.0; SD = 3.0
Sex	Female = 14; Male = 8
Breed	Goldendoodle = 3; Golden Retriever = 2; Poodle mix = 2; Labrador Retriever mix = 2; Labrador Retriever = 1; Standard Poodle = 1; Miniature Poodle = 1; Toy Poodle = 1; Alaskan Husky = 1; Australian Shepherd = 1; Australian Shepherd mix = 1; Beagle = 1; Brittany Spaniel = 1; Chihuahua = 1; Great Dane = 1; Rough Collie = 1; Whippet mix = 1

#### Ethical Note

All children, parents, and dogs participated on a voluntary basis. Written informed consent was obtained from the parents/guardians of all participants, and assent was obtained from all of the children explicitly indicating their understanding and desire to participate in the research. The Institutional Review Board (IRB) and Institutional Animal Care and Use Committee (IACUC) of Oregon State University approved all methods and procedures for this study.

#### Intervention

Families were recruited from the northwestern region of the United States to participate in a randomized control study involving synchronous activity-based AAI for youth with developmental disabilities. For the current study we focused on 24 child-dog dyads randomly assigned to one of two AAI groups [12 participated in a “Do As I Do” dog training intervention (Fugazza, 2014) and 12 in a dog walking intervention]. Both the dog training and dog walking interventions were conducted in ten 1-h sessions on a university campus. During the summer the interventions were offered 5 days per week for 2 weeks and during the school year the interventions were offered 2 days per week for 5 weeks. A pair of trained research assistants worked with each participant-dog dyad for all ten sessions, coaching the child on how to train their dog on the objectives of the study group to which they were assigned (i.e., “Do As I Do” training or leash walking). At home participants were instructed to walk their dog for 30 min and work on the intervention training skills with their dog for 5 min every day as homework throughout the course of the intervention. Assessments were conducted with all participant-dog dyads during the week prior to the start of the intervention and the week after the end of the intervention.

#### Secure Base Test

As in the pilot study, the SBT was used to evaluate the attachment behavior of the family dog toward the child participant at the initial and follow-up assessments. The SBT was also used to evaluate the attachment behavior exhibited by the dog toward an adult owner/parent for comparison to the dog’s behavior toward the child. The same methodology was used across both studies.

#### Behavior Coding

As in the pilot study, all assessments were video recorded and the return phase was viewed by two coders who independently categorized the dog’s behavior using an established ethogram for canine attachment style categories (Table 2). There was 68.6% independent inter-rater agreement for attachment style categorization (binomial probability, *p* < 0.001). Categorization disagreements were then jointly reviewed to come to consensus for the final attachment style designation. The broader categorization of secure or insecure attachment, indicating the presence or absence of the Secure Base Effect, was the primary focus in this study.

### Results and Discussion

Similar to what was observed in the pilot study, nine dogs exhibited a secure attachment to the child and 18 dogs exhibited a secure attachment to the parent. All nine dogs with a secure attachment to the child also had a secure attachment to the parent (Binomial Test, *p* = 0.004). No dogs with an insecure attachment to the parent (*n* = 6) had a secure attachment to the child (Binomial Test, *p* = 0.03).

At the follow-up assessment, 18 dogs had a secure attachment to the child. This was a statistically significant increase in the number of secure attachment bonds between dog and child participants when comparing pre- and post-intervention attachment styles (Fisher’s Exact Test, *p* = 0.02). In addition, 15 of those 18 dogs also had a secure attachment to the parent (Binomial Test, *p* = 0.008), suggesting that while some dogs can form a secure attachment to the child partner post-intervention without having a secure attachment to the primary caregiver (in this case 3 dogs), a secure attachment to the parent still appeared to be a significant predictive variable. No dogs shifted from a secure attachment style toward the child pre-intervention to an insecure attachment style post-intervention, again supporting prior findings that participation in this kind of AAI typically has a neutral to beneficial impact on the dog-human bond (Tepfer et al., 2017).

## General Discussion

Research indicates that the bonds between dogs and adult owners can fulfill the criteria of an attachment bond (Topál et al., 1998; Palmer and Custance, 2008; Mariti et al., 2013), and some studies have gone on to categorize dogs into formal attachment styles (Schöberl et al., 2016; Thielke et al., 2017). The current findings demonstrate that, in at least some cases, dogs can also form a secure attachment bond to a child in the household. Furthermore, in these two studies, the attachment quality between dog and child was predicted by the attachment style of the dog toward a primary adult caregiver. Participation in a joint activity-based AAI also helped improve the attachment security of the dog-child bond in some cases. These findings shed light on how dogs may perceive children in their environment, in what ways relationships with other bonded humans influences this perception and how participation in joint interventions with a child partner can impact the dog-child bond.

In many households the primary caregiver and attachment figure of the family dog is an adult owner (Hall et al., 2016). Therefore, it is not surprising that more secure attachments were observed between the dogs and adult owners than between dogs and children during initial assessments. However, it is common for both humans and dogs to have multiple attachment figures (Topál et al., 1998; Howes and Spieker, 2008; Kurdek, 2009; Mariti et al., 2011), and the current findings demonstrate that secure attachments between a dog and a child in the same household may exist prior to, or develop during, a child-focused AAI. Moreover, the present results support prior evidence from the human literature that the establishment of a secure attachment style to primary caregivers can influence the strength and security of attachments formed with other individuals (Simpson, 1990; Maccoby, 1992; Smyke et al., 2010), in this case children in the same household. While more research is needed to determine the full range of variables that may contribute to secure attachment development between dogs and children in the household, it seems promising that AAIs developed with joint participation and mutual well-being in mind have the potential to improve attachment bonds between human and animal participants.

Evidence of secure attachment development between dog-child AAI partners has a number of important applied implications. For example, secure attachments have been shown to have a wide range of benefits including stress reduction, increased exploration and persistence, improved executive function, and a reduction of behavior problems in dogs and humans (Bowlby, 1982; de Ruiter and van IJzendoorn, 1993; Cooper et al., 1998; Horn et al., 2013; Bernier et al., 2015). When the child becomes a secure base for a dog AAI partner, this could also enhance the dog’s sense of security to engage in the environment alongside the child (Julius et al., 2013), possibly improving the animal’s welfare, focus, or performance in the AAI (Wanser and Udell, 2019). Changes in the dog’s behavior may increase a child’s perceptions of their own attachment toward their dog, which can also have a positive effect on wellbeing and AAI outcomes (Hall et al., 2016; Wanser et al., 2019). For example, the dog may seek out the child more for interaction at home when the parent is absent/unavailable, fostering increased interactions and greater feelings of responsibility and companionship for the child. Additional research is needed to evaluate these possibilities, and to expand on the current findings. Future research should also evaluate additional behavioral differences in securely attached dogs toward AAI partners who double as caretakers, as benefits may extend beyond those already identified in therapeutic settings with visiting therapy dogs (Wanser and Udell, 2019). However, the knowledge that at least some AAIs can have a beneficial impact on the dog-human relationship – and more specifically the dog-child relationship (with no evidence of a negative impact identified in the current study) – is a promising finding that supports the One-Health mission of many AAI efforts. More research will help improve our understanding of how the dog-human bond may influence AAI outcomes, to identify ways to maximize the health and wellbeing of animal and human participants, as well as to improve human-animal interactions in AAI settings. Furthermore, research focused on the child-dog bond may provide important insights into similarities and differences in the ways dogs and children perceive one another (compared with dog-adult human relationships), interact together, and in some cases, serve as support to one another within the home and in AAI settings.

## Data Availability Statement

The raw data supporting the conclusions of this article will be made available by the authors, without undue reservation.

## Ethics Statement

The studies involving human participants were reviewed and approved by the Institutional Review Board (IRB) of Oregon State University. Written informed consent to participate in this study was provided by the participants’ legal guardian/next of kin. The animal study was reviewed and approved by the Institutional Animal Care and Use Committee (IACUC) of Oregon State University. Written informed consent was obtained from the owners for the participation of their animals in this study.

## Author Contributions

MU and MM conceived and planned both studies. SW, MU, and MM carried out the pilot study. SW, AS, MU, and MM carried out the AAI Experiment. SW and MU analyzed the data and wrote the manuscript with contributions from AS and MM. All authors contributed to the article and approved the submitted version.

## Conflict of Interest

The authors declare that the research was conducted in the absence of any commercial or financial relationships that could be construed as a potential conflict of interest. The reviewer AB declared a past co-authorship with one of the authors MU to the handling editor.

## References

[B1] Ainsworth M. D. (1989). Attachments beyond infancy. *Am. Psychol.* 44 709–716. 10.1037/0003-066x.44.4.709 2729745

[B2] Barker S. Barker R. (1988). The human-canine bond: closer than family ties? *J. Mental Health Counsel.* 10 46–56.

[B3] Bennett P. C. Rohlf V. I. (2007). Owner-companion dog interactions: relationships between demographic variables, potentially problematic behaviours, training engagement and shared activities. *Appl. Animal Behav. Sci.* 102 65–84. 10.1016/j.applanim.2006.03.009

[B4] Bernier A. Beauchamp M. H. Carlson S. M. Lalonde G. (2015). A secure base from which to regulate: attachment security in toddlerhood as a predictor of executive functioning at school entry. *Dev. Psychol.* 51 1177–1189. 10.1037/dev0000032 26192039

[B5] Bowlby J. (1958). The nature of the child’s tie to his mother. *Int. J. Psychoanalysis* 39 350–373. 13610508

[B6] Bowlby J. (1982). *Attachment and Loss. Vol. 1. Attachment*, 2nd Edn New York, NY: Basic Books.

[B7] Chitic V. Rusu A. S. Szamoskozi S. (2020). The effects of animal assisted therapy on communication and social skills: a meta-analysis. *Trans. J. Psychol.* 13 1–17.

[B8] Cohen S. P. (2002). Can pets function as family members? *Western J. Nurs. Res.* 24 621–638. 10.1177/019394502320555386 12365764

[B9] Cooper M. L. Shaver P. R. Collins N. L. (1998). Attachment styles, emotion regulation, and adjustment in adolescence. *J. Pers. Soc. Psychol.* 74 1380–1397. 10.1037/0022-3514.74.5.1380 9599450

[B10] de Ruiter C. van IJzendoorn M. H. (1993). Attachment and cognition: a review of the literature. *Int. J. Educ. Res.* 19 525–540.

[B11] Fugazza C. (2014). *Do As I Do: Using Social Learning to Train Dogs.* Wenatchee, WA: Dogwise.

[B12] Gácsi M. Topál J. Miklósi Á Dóka A. Csányi V. (2001). Attachment behavior of adult dogs (Canis familiaris) living at rescue centers: forming new bonds. *J. Comp. Psychol.* 115 423–431. 10.1037/0735-7036.115.4.423 11824906

[B13] Garrity T. F. Stallones L. Marx M. B. Johnson T. P. (1989). Pet ownership and attachment as supportive factors in the health of the elderly. *Anthrozoos* 3 35–44. 10.2752/089279390787057829

[B14] Hall N. J. Liu J. Kertes D. A. Wynne C. D. L. (2016). Behavioral and self-report measures influencing children’s reported attachment to their dog. *Anthrozoos* 29 137–150. 10.1080/08927936.2015.1088683 28066130 PMC5214578

[B15] Harlow H. F. (1958). The nature of love. *Am. Psychol.* 13 673–685. 10.1037/h0029383 4984312

[B16] Horn L. Huber L. Range F. (2013). The importance of the secure base effect for domestic dogs - evidence from a manipulative problem-solving task. *PLoS One* 8:e65296. 10.1371/journal.pone.0065296 23734243 PMC3667003

[B17] Howes C. Spieker S. (2008). “Attachment relationships in the context of multiple caregivers,” in *Handbook of Attachment: Theory, Research, and Clinical Applications*, eds Cassidy J. Shaver P. R. (New York, NY: The Guilford Press), 317–332.

[B18] Hu M. Zhang P. Leng M. Li C. Chen L. (2018). Animal-assisted intervention for individuals with cognitive impairment: a meta-analysis of randomized controlled trials and quasi- randomized controlled trials. *Psychiatry Res.* 260 418–427. 10.1016/j.psychres.2017.12.016 29268204

[B19] Jones M. G. Rice S. M. Cotton S. M. (2019). Incorporating animal-assisted therapy in mental health treatments for adolescents: systematic review of canine assisted psychotherapy. *PLoS One* 14:e0210761. 10.1371/journal.pone.0210761 30653587 PMC6336278

[B20] Julius H. Beetz A. Kotrschal K. Turner D. Uvnäs-Moberg K. (2013). *Attachment to Pets: an Integrative View of Human-Animal Relationships with Implications for Therapeutic Practice.* Cambridge, MA: Hogrefe Publishing

[B21] Kurdek L. A. (2009). Pet dogs as attachment figures for adult owners. *J. Fam. Psychol.* 23 439–446. 10.1037/a0014979 19685978

[B22] Leonardi R. J. Buchanan-Smith H. M. McIvor G. Vick S.-J. (2017). “You think you’re helping them, but they’re helping you too”: experiences of Scottish male young offenders participating in a dog training program. *Int. J. Environ. Res. Public Health* 14 1–27. 10.3390/ijerph14080945 PMC5580647 28829389

[B23] Linder D. E. Mueller M. K. Gibbs D. M. Alper J. A. Freeman L. M. (2018). Effects of an animal-assisted intervention on reading skills and attitudes in second grade students. *Early Childhood Educ. J.* 46 323–329. 10.1007/s10643-017-0862-x

[B24] Maccoby E. E. (1992). The role of parents in the socialization of children: an historical overview. *Dev. Psychol.* 28 1006–1017. 10.1037/0012-1649.28.6.1006

[B25] Mariti C. Carlone B. Borgognini-Tarli S. Presciuttini S. Pierantoni L. Gazzano A. (2011). Considering the dog as part of the system: studying the attachment bond of dogs toward all members of the fostering family. *J. Vet. Behav.* 6 90–91. 10.1016/j.jveb.2010.08.026

[B26] Mariti C. Ricci E. Zilocchi M. Gazzano A. (2013). Owners as a secure base for their dogs. *Behaviour* 150 1275–1294. 10.1163/1568539x-00003095

[B27] Meyer I. Forkman B. (2014). Dog and owner characteristics affecting the dog-owner relationship. *J. Vet. Behav.* 9 143–150. 10.1016/j.jveb.2014.03.002

[B28] O’Haire M. E. (2017). Research on animal-assisted intervention and autism spectrum disorder, 2012-2015. *Appl. Dev. Sci.* 3 200–216. 10.1080/10888691.2016.1243988 31080343 PMC6510492

[B29] Overall K. L. Love M. (2001). Dog bites to humans - demography, epidemiology, injury, and risk. *J. Am. Vet. Med. Assoc.* 218 1923–1934. 10.2460/javma.2001.218.1923 11417736

[B30] Palmer R. Custance D. (2008). A counterbalanced version of Ainsworth’s Strange Situation procedure reveals secure-base effects in dog-human relationships. *Appl. Animal Behav. Sci.* 109 306–319. 10.1016/j.applanim.2007.04.002

[B31] Parthasarathy V. Crowell-Davis S. L. (2006). Relationship between attachment to owners and separation anxiety in pet dogs (Canis lupus familiaris). *J. Vet. Behav.* 1 109–120. 10.1016/j.jveb.2006.09.005

[B32] Rooney N. J. Bradshaw J. W. (2002). An experimental study of the effects of play upon the dog-human relationship. *Appl. Animal Behav. Sci.* 75 161–176. 10.1016/s0168-1591(01)00192-7

[B33] Schöberl I. Beetz A. Solomon J. Wedl M. Gee N. Kotrschal K. (2016). Social factors influencing cortisol modulation in dogs during a strange situation procedure. *J. Vet. Behav.* 11 77–85. 10.1016/j.jveb.2015.09.007

[B34] Serpell J. Barrett P. (1995). *The Domestic Dog: its Evolution, Behavior and Interactions with People.* New York, NY: Cabridge University Press.

[B35] Simpson J. A. (1990). Influence of attachment styles on romantic relationships. *J. Pers. Soc. Psychol.* 60 861–869.

[B36] Smyke A. T. Zeanah C. H. Fox N. A. Nelson C. A. Guthrie D. (2010). Placement in foster care enhances quality of attachment among young institutionalized children. *Child Dev.* 81 212–223. 10.1111/j.1467-8624.2009.01390.x 20331663 PMC4098033

[B37] Stewart R. B. (1983). Sibling attachment relationships: child-infant interactions in the strange situation. *Dev. Psychol.* 19 192–199. 10.1037/0012-1649.19.2.192

[B38] Tepfer A. Ross S. MacDonald M. Udell M. A. R. Ruaux C. Baltzer W. (2017). Family dog-assisted adapted physical activity: a case study. *Animals (Basel)* 7 35. 10.3390/ani7050035 28448430 PMC5447917

[B39] Thielke L. E. Rosenlicht G. Saturn S. R. Udell M. A. R. (2017). Nasally-administered oxytocin has limited effects on owner-directed attachment behavior in pet dogs (Canis lupus familiaris). *Front. Psychol.* 8:1699. 10.3389/fpsyg.2017.01699 29033879 PMC5626864

[B40] Thielke L. E. Udell M. A. R. (2019). Evaluating cognitive and behavioral outcomes in conjunction with the secure base effect for dogs in shelter and foster environments. *Animals* 9 932. 10.3390/ani9110932 31703387 PMC6912329

[B41] Thielke L. E. Udell M. A. R. (2020). Characterizing human–dog attachment relationships in foster and shelter environments as a potential mechanism for achieving mutual wellbeing and success. *Animals* 10 67. 10.3390/ani10010067 31905973 PMC7023214

[B42] Topál J. Miklósi Á Csányi V. Dóka A. (1998). Attachment behavior in dogs (Canis familiaris): a new application of Ainsworth’s (1969) strange situation test. *J. Comp. Psychol.* 112 219–229. 10.1037/0735-7036.112.3.219 9770312

[B43] Vitale K. R. Behnke A. C. Udell M. A. R. (2019). Attachment bonds between domestic cats and humans. *Curr. Biol.* 29 R864–R865. 31550468 10.1016/j.cub.2019.08.036

[B44] Wanser S. H. Udell M. A. R. (2019). Does attachment security to a human handler influence the behavior of dogs who engage in animal assisted activities? *Appl. Animal Behav. Sci.* 210 88–94. 10.1016/j.applanim.2018.09.005

[B45] Wanser S. H. Vitale K. R. Thielke L. E. Brubaker L. Udell M. A. R. (2019). Spotlight on the psychological basis of childhood pet attachment and its implications. *Psychol. Res. Behav. Manag.* 2019 469–479. 10.2147/prbm.s158998 31303801 PMC6610550

[B46] Yin S. (2011). *Dog Bite Prevention: Dogs Bite When Humans Greet Inappropriately*. Cattledog Publishing: The Art and Science of Animal Behavior Available online at: https://drsophiayin.com/blog/entry/dog-bite-prevention-dogs-bite-whenhumans-greet-inappropriately/

